# Design, Modeling, Self-Calibration and Grasping Method for Modular Cable-Driven Parallel Robots

**DOI:** 10.3390/s26072204

**Published:** 2026-04-02

**Authors:** Wanlin Mai, Yonghe Wang, Zhiquan Yang, Bin Zhu, Lin Liu, Jianqing Peng

**Affiliations:** 1 School of Intelligent Systems Engineering, Shenzhen Campus of Sun Yat-sen University, Shenzhen 518107, China; maiwlin@mail2.sysu.edu.cn (W.M.); wangyh577@mail2.sysu.edu.cn (Y.W.); yangzhq33@mail2.sysu.edu.cn (Z.Y.); zhub26@mail.sysu.edu.cn (B.Z.); 2 School of Advanced Manufacturing, Sun Yat-sen University, Shenzhen 518107, China; 3 Guangdong Provincial Key Laboratory of Fire Science and Technology, Guangzhou 510006, China

**Keywords:** modular cable-driven parallel robot, kinematic modeling, self-calibration, RGB-D visual grasping

## Abstract

Cable-driven parallel robots (CDPRs) are attractive for large-space manipulation because of their lightweight structure, large workspace, and reconfigurability. However, existing systems still face three practical challenges: limited modularity of the mechanical architecture, repeated calibration after reconfiguration, and insufficient integration between visual perception and grasp execution. To address these issues, this paper presents a modular cable-driven parallel robot (MCDPR), together with its kinematic modeling, vision-based self-calibration, and visual grasping methods. First, a modular mechanical architecture is developed in which the drive, sensing, and cable-guiding functions are integrated to support rapid assembly/disassembly, convenient debugging, and cable anti-slack operation. Second, a pulley-considered multilayer kinematic model is established, and a vision-based self-calibration method is proposed to identify the structural parameters after assembly using onboard sensing and AprilTag observations, thereby reducing the number of recalibrations required during robot operation after reconfiguration. Third, a vision-guided bin-picking method is developed by combining RGB-D perception, coordinate transformation, and the calibrated robot model. Simulation and prototype experiments are conducted to validate the proposed system. A software/hardware combined validation framework is established, in which the CoppeliaSim-based simulation and the hardware prototype are used together to verify the proposed design and methods. In simulation, self-calibration reduces the Euclidean grasping position error from 0.371 mm to 0.048 mm and the orientation error from 0.071° to 0.004°. In experiments, the relative position error is reduced by 58.33% after self-calibration.

## 1. Introduction

Cable-driven parallel robots (CDPRs) have attracted increasing attention because of their lightweight structure, large workspace, and reconfigurability [1,2,3,4]. These features make CDPRs promising for applications such as large-space manipulation, high-speed transportation, and medical assistance. However, despite these advantages, the practical use of CDPRs still faces several challenges, especially in systems that need to be assembled, adjusted, and reused in different task scenarios.

A conventional CDPR usually consists of a fixed frame and a moving platform. Although this configuration can provide high-speed motion with a relatively light end platform, the overall system is still strongly dependent on the frame structure, which limits its flexibility in practical deployment [5]. In particular, when the robot needs to be reassembled or reconfigured, the rebuilding of the frame and the rearrangement of cable routing usually require considerable time and effort. For this reason, modular design has gradually become an important direction in CDPR research [6]. Nevertheless, many existing systems still adopt functionally separated architectures, in which the drive, guiding, and sensing units are not organized as relatively independent modules [7]. As a result, rapid reconstruction and convenient debugging remain difficult to achieve in practice.

In recent years, extensive studies have been carried out on the kinematic modeling of CDPRs. Since the forward kinematics of CDPRs generally involves solving nonlinear constraint equations, different methods have been proposed, including homotopy methods, interval analysis, optimization-based methods, and analytical solutions [8,9,10,11,12,13,14,15,16]. For example, Carricato et al. [10] studied the forward kinematics of under-constrained CDPRs using a homotopy-based approach, and Martin-Parra et al. [11] used interval analysis to solve similar problems. These studies provide an important basis for the modeling and analysis of CDPRs. However, most of them are developed for fixed robot configurations, and the influence of modular reconstruction on the modeling process is rarely discussed.

Trajectory tracking and control are also important topics in CDPR research. Existing studies have improved tracking performance by optimizing cable configuration, workspace distribution, or tension allocation [17,18,19,20,21,22,23]. These methods have shown good performance in specific systems, especially in improving motion stability and control accuracy. However, most of them are still established on the assumption that the robot structure is fixed. For modular or frequently reconfigured CDPRs, the coupling between structural change and control performance has not been sufficiently considered.

Calibration is another key issue affecting the accuracy of CDPRs. Existing calibration methods can generally be divided into external calibration and self-calibration. External calibration methods usually rely on high-precision measurement devices, such as inclinometers, localizers, or laser-based equipment [24,25,26]. In contrast, self-calibration methods estimate structural parameters by using onboard sensing information, such as encoders and force sensors [27,28,29,30,31,32,33]. For example, Joshi et al. [24] proposed a calibration method for 6-DOF CDPRs using inclinometers, and Miermeister et al. [26] developed an automatic calibration method based on internal sensors. Although these studies have achieved good results, existing methods are either dependent on external equipment or designed for specific robot configurations. This limits their applicability to modular CDPRs that need repeated assembly and reconfiguration.

Modular design has been recognized as an effective way to improve the flexibility and adaptability of CDPRs. Some studies have explored modular CDPRs in practical applications [34,35,36]. For instance, Iturralde et al. [34] developed a CDPR system with interchangeable end-effectors for construction tasks. These studies show the potential of modular design in expanding application scenarios and improving deployment efficiency. However, most existing work mainly focuses on mechanical modularity itself, while the integration of modular design with calibration and task execution has not been sufficiently studied.

To clarify the relationship between representative existing studies and the present work, Table 1 summarizes the existing literature from the perspectives of research focus, system characteristics, and limitations related to modular or reconfigurable CDPRs.

From the above review, several research gaps can be identified. First, existing CDPR systems still lack a relatively unified modular architecture that can support both rapid reconfiguration and stable cable routing. Second, current calibration methods are either dependent on external devices or limited to particular configurations, which makes them less suitable for repeatedly reconfigured systems. Third, the connection between calibration results and downstream task execution, such as vision-guided grasping, is still not well established in modular CDPR systems.

To address these issues, this paper proposes a modular cable-driven parallel robot (MCDPR) and studies its mechanical design, kinematic modeling, vision-based self-calibration, and visual grasping method. The main contributions of this paper are summarized as follows:

1. A modular MCDPR prototype is developed, in which the drive, sensing, cable-guiding, and tension-maintenance functions are integrated to support rapid assembly/disassembly, convenient debugging, and anti-slack operation.
2. A pulley-considered multilayer kinematic model is established for the proposed MCDPR, and a vision-based self-calibration method is further developed to identify the structural parameters after assembly. Unlike calibration methods that rely on external instruments or are developed for fixed configurations, the proposed method is intended for repeated assembly scenarios and can reduce the number of recalibrations required during robot operation after system replacement.
3. A vision-guided bin-picking method is constructed by combining RGB-D perception and the calibrated kinematic model, so that the calibration results can be directly used for grasp execution within the modular CDPR system rather than being treated as an isolated calibration procedure.
4. A simulation platform and a hardware prototype are built for validation, and experiments on self-calibration, trajectory tracking, tension maintenance, and grasping are conducted to evaluate the proposed system.

The remainder of this paper is organized as follows. Section 2 introduces the modular mechanical design of the MCDPR. Section 3 presents the forward and inverse kinematic models. Section 4 describes the vision-based self-calibration method. Section 5 introduces the vision-guided bin-picking method. Section 6 reports the simulation and experimental results, and Section 7 concludes the paper.

## 2. Modular Structure Design

**Figure 1:**
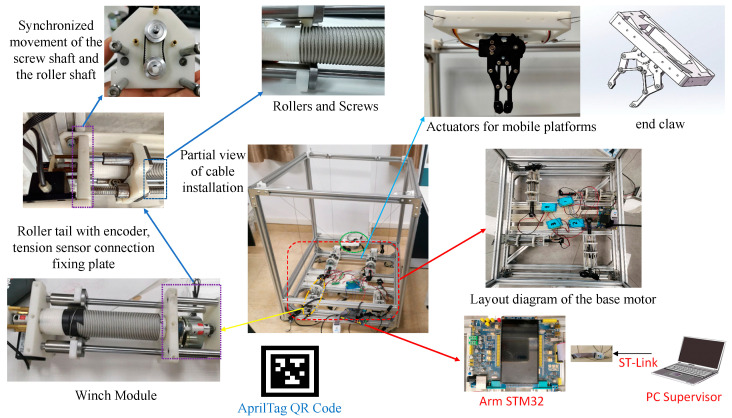
The prototype of the MCDPR.

This section describes the mechanical design of the proposed MCDPR with emphasis on reconfiguration-oriented integration. The arrangement of the drive units, pulleys, sensors, and redundant cable is designed to improve assembly convenience, reduce cable slack risk, and preserve a usable workspace for vision-guided manipulation. As shown in Figure 1, the moving platform of the MCDPR is driven by four cables to achieve wide-range motion. The positive cable tension not only actuates the platform but also helps maintain its stability under load variation. The prototype mainly consists of a mechanical system, a controller, and sensors. The mechanical system includes the frame, cables, drive unit, and end-effector carrying the gripper and camera. The controller is based on an STM32 (STM32F103C8T6, ALIENTEK, Guangzhou, China) development board and a host computer, and the sensing system includes vision, angle, and tension sensors. In addition, high-precision force sensors and external encoders are integrated into the winch module to support cable tension measurement and drum-rotation feedback.

Each cable can be modeled as an SPS kinematic chain; therefore, the MCDPR can be regarded as a multi-chain closed-loop system. To preserve the upper workspace for manipulation, the motors, rollers, actuators, and controllers are arranged at the bottom of the frame, and the cables are routed upward through pulley groups to the fixed anchor seats. In this way, the upper part of the frame is mainly used as the operating space, whereas the lower part accommodates the drive and control hardware.

The frame of the MCDPR is a rectangular structure constructed from standard 3030 aluminum profiles. The internal workspace of the current prototype is approximately 620 mm × 620 mm × 730 mm. In addition, a high-precision AprilTag marker is attached to the partition board of the MCDPR to support the vision-based self-calibration process and trajectory-tracking-related pose observation. This size was selected to support vision observation, trajectory tracking, and grasping experiments, while remaining compatible with laboratory assembly, structural stiffness, cable routing, and sensor installation.

The present prototype serves as a small-scale validation platform for a scalable modular design route [20]. Owing to the modularized integration of the drive, sensing, and guiding functions, the same design logic can be extended to larger frames by adjusting the structural members, anchor-point layout, and cable lengths.

**Figure 2:**
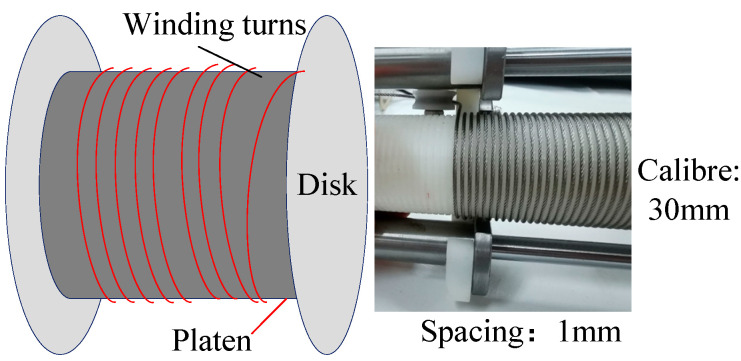
Partial view of cable installation.

To reduce the space occupied by cable winding, a winch is used as the reel, as shown in Figure 2. This design helps control the number of cable turns on the drum, so that the cable can be wound neatly and tightly, thereby avoiding overlap and irregular stacking.

**Figure 3:**
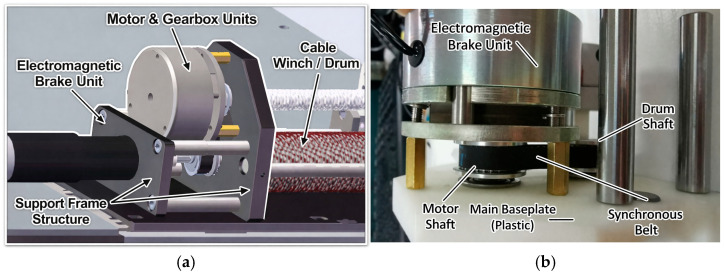
Electromagnetic brake design. (**a**) Simulation model. (**b**) Physical prototype.

As shown in Figure 3, the motor output is transmitted through the gearbox and then connected to the drum shaft by elastic coupling, so that the motor rotation drives the drum directly. Ball screws and guide rods are arranged around the drum to support the pulleys and linear bearings. In addition, a synchronous belt mechanism connects the screw shaft and drum shaft to maintain synchronized rotation, while a brake is mounted at the end of the screw shaft to stop the entire drive unit when needed.

**Figure 4:**
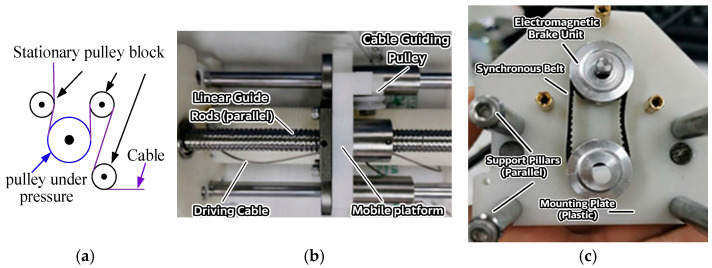
Schematic diagram of the screw. (**a**) Tension sensor winding. (**b**) Assembly drawing of winch screw. (**c**) Synchronization diagram of screw shaft and roller shaft.

The motor shafts are connected to the corresponding drum shafts through couplings, and the cables are routed out through the pulley set at the bottom of the winch. At the four upper corners of the frame, fixed anchor seats and guide pulleys are arranged symmetrically. The cables are led upward from the bottom pulley group to the guide pulleys and then redirected to the fixed anchor pulleys. This layout keeps the cable segment from the bottom pulley to the guide pulley approximately vertical, while maintaining the cable segment near the fixed anchor pulley approximately parallel to the horizontal plane, thereby reducing kinematic error. A single-pulley tension sensor is adopted so that the cable angle at the stressed pulley remains fixed during measurement, which helps improve the consistency of tension sensing. In addition, the ball screw is selected as the element rotating synchronously with the drum to reduce transmission loss. Figure 3 and Figure 4 illustrate the assembly details and the corresponding mechanical design considerations of the proposed system. The integrated transmission path improves the compactness of the drive module, the guided cable-routing layout helps maintain a stable cable path and reduce kinematic uncertainty, and the tension sensor, encoder, and redundant cable arrangement supports anti-slack control. In particular, the cable is kept taut through a preset pre-tightening force, which helps prevent slack and cable derailment during operation. Unlike conventional modular CDPR designs that mainly emphasize structural replaceability, the proposed architecture integrates the drive, sensing, cable-guiding, and anti-slack functions within the same framework, which supports reconfiguration as well as the subsequent calibration and task execution [20].

Because cables can only exert tensile forces, maintaining stable and fully constrained motion in an MCDPR is challenging. A common solution is to introduce redundant cables to improve stability, maintain controllability, and avoid cable slack [17,18]. In the prototype designed in this study, four cables are used: cables 1–3 generate the main spatial motion, while cable 4 acts as a redundant actuator for tension maintenance [23].

**Figure 5:**
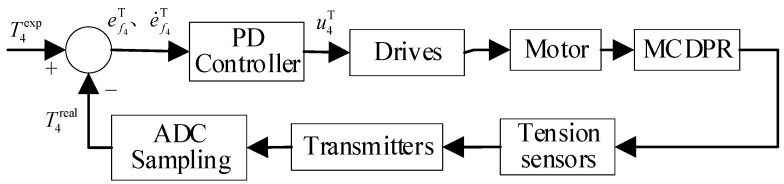
Block diagram of constant force control for redundant cable.

The variable definitions in this article are shown in Table 2. Cables 1~3 are utilized to enable the robot to achieve three DOFs of space motion within a workspace measuring approximately 620 mm × 620 mm × 730 mm. Cable 4 serves as a redundant actuator. As illustrated in Equation (1) and Figure 5, a PD control strategy is applied to maintain constant tension in cable 4, ensuring that all cables remain taut in real time throughout the robot’s motion [37]:

(1) $$u_{4}^{T}=K_{p}^{T}\cdot e_{f_{4}}^{T}+K_{d}^{T}\cdot {\dot{e}}_{f_{4}}^{T}$$

where $K_{p}^{T}$, $K_{d}^{T}$ are the proportionality and differential constants, respectively, $e_{f_{4}}^{T}$ and ${\dot{e}}_{f_{4}}^{T}$ are the tension error of cable 4 and the differential of the tension error, respectively, and $u_{4}^{T}$ is the amount of drive current control for cable 4.

In this study, the PD law for cable 4 is mainly used to maintain the desired tension range during motion and suppress cable slack [37]. Since cable 4 is mainly used to maintain the tension state of the system during motion, the corresponding control objective is to keep the cable tension within the desired range and suppress slack. The disturbance experiment in Section 6.2.4 further shows that this control law can maintain the cable tension within the required range on the current prototype.

## 3. Kinematic Modeling of an MCDPR

This section presents the kinematic modeling of the proposed MCDPR. We first derive the inverse kinematic relationship from the drum rotation, cable routing, and moving platform geometry, and then formulate the forward kinematics as an optimization problem. These models provide the basis for both structural calibration and motion execution in the later sections.

### 3.1. Inverse Kinematic Equations

Since the cable is wrapped around the drum without any reducer, the transmission ratio is 1:1. The variation in cable length is realized by the rotation of the drum in the winch driven by the motor, so the relationship between the length of the *i*th cable (i.e., $l_{i}$) and the angle of the *i*th drum (i.e., $\theta_{i}$) can be described as follows:

(2) $$l_{i}=R_{\mathrm{wih}}\cdot \theta_{i}$$

For the whole MCDPR, Equation (2) can be written in matrix form as follows:

(3) $$L=R_{\mathrm{wih}}\cdot \Theta$$

where $\Theta ={\left[\theta_{1},\cdots ,\theta_{N_{r}}\right]}^{T}$, $L={\left[l_{1},\cdots ,l_{N_{r}}\right]}^{T}$, and $R_{\mathrm{wih}}$ is the radius of the winding drum of the winch module.

Assuming that the initial angle of the *i*th drum is $\theta_{i}^{\left(0\right)}$, the mapping relationship between the initial cable length $l_{i}^{\left(0\right)}$ of the *i*th cable and the corresponding $\theta_{i}^{\left(0\right)}$ can be expressed as follows:

(4) $$l_{i}^{\left(0\right)}=R_{\mathrm{wih}}\cdot \theta_{i}^{\left(0\right)}$$

For the entire MCDPR, Equation (4) can be further rewritten as follows:

(5) $$L^{\left(0\right)}=R_{\mathrm{wih}}\cdot \Theta^{\left(0\right)}$$

where $\Theta^{\left(0\right)}={\left[\theta_{1}^{\left(0\right)},\cdots ,\theta_{N_{r}}^{\left(0\right)}\right]}^{T}$ and $L^{\left(0\right)}={\left[l_{1}^{\left(0\right)},\cdots ,l_{N_{r}}^{\left(0\right)}\right]}^{T}$.

Thus, the mapping relationship between the anchor point seat, the moving platform, and the drum angle of rotation can be expressed as follows:

(6) Rwih·Θ=BiwCi⏜+Liw2

where BiwCi⏜ denotes the length of the arc-shaped cable segment and Liw2 denotes the length of the straight cable segment.

Then, the kinematic model of the MCDPR can be expressed as follows:

(7) fiAm,iBw,iPmw,θi,Rwih,Rp=0

where Pmw=xm,ym,zm,αm,βm,γmT and $R_{p}$ is the radius of the pulley.

Equation (7) can be further written in the form of a matrix, namely, the following:

(8) fAim,Biw,Pmw,Θ,Rwih,Rp=f1Aim,Biw,Pmw,θi,Rwih,Rpf2Aim,Biw,Pmw,θi,Rwih,Rp⋮fNrAim,Biw,Pmw,θi,Rwih,Rp=O

The combination of Equation (2) and Equation (8) can be rewritten as follows:

(9) fAm,iBw,iPmw,L,Rp=O

By moving all terms on the left-hand side of Equation (9) that are independent of L to the right-hand side, the inverse kinematic equation of the MCDPR can be written as follows:

(10) L=finvAm,iBw,iPmw,Rp

Due to $L={\left[l_{1},\cdots ,l_{N_{r}}\right]}^{T}$, we obtain the following:

(11) li=fiinvAm,iBw,iPmw,Rp

Assuming that the mass of the cable is not taken into account and that the material of the cable is considered to be homogeneous, the tension of the cable does not change steeply, and the velocity of the cable is equal at all points [15]. Thus, the inverse kinematics equation of the velocity level of the MCDPR can be expressed as follows [16]:

(12) L˙=JL·P˙mw

where $J_{L}$ is the Jacobi of the velocity of the cable length to the velocity of the end-effector.

### 3.2. Positive Kinematic Equations

Based on Equation (11), let Pmkw denote the fitted value for the *k*th iteration of the moving platform pose (i.e., Pmw). Then, the corresponding fitted value for the cable length is lik=fiinvAim,Bim,Pmkw,Rp. Assuming that the actual cable length is lir, the optimization function of the cable length can be expressed as follows:

(13) FiAm,iBw,iPmkw,lir,Rp=lir−lik=lir−fiinvAm,iBw,iPmkw,Rp

Expanding Equation (11) yields the following:

(14) F1Am,1Bw,1Pm1w,lr,1RpF2Am,2Bw,2Pm2w,lr,2Rp⋮FNrAm,NrBw,NrPmNrw,lNrr,Rp=l1r−f1invAm,1Bm,1Pm1w,Rpl2r−f2invAm,2Bm,2Pm2w,Rp⋮lNrr−fNrinvAm,NrBm,NrPmNrw,Rp

Equation (14) can be further rewritten as follows:

(15) FAm,Bw,P˜w,mLr,Rp=Lr−finvAm,Bw,P˜mw,Rp

where Lr=lr,1lr,2⋯,lNrrT is the length matrix of the actual cable, Am=A1m;⋯,ANrm, Bw=B1w;⋯,BNrw, and P˜mw=Pm1w;⋯;PmNrw.

Further, the positive kinematics optimization equation for the MCDPR can be expressed as follows:

(16) Pf=argminPmkw∈UpFAm,Bw,P˜mw,Lr,Rp

where $P_{f}$ is the iterative optimization solution and $U_{p}$ is the set of reachable workspaces for the moving platforms.

The optimization problem shown in Equation (14) can be solved by the Newton iterative method [10,13], in which the choice of the initial value of iteration Pm0w directly affects the subsequent solution efficiency and whether it converges to the global optimum or not.

## 4. Self-Calibration Method for CDPRs

This section describes the self-calibration method of the MCDPR. The key idea is to combine cable length observations from encoders with pose observations from the onboard vision system, so that the structural parameters after assembly can be identified without relying on large external measuring devices.

### 4.1. Definition of Calibration Variables

An MCDPR is over-constrained when the number of driving cables exceeds the DOFs of the moving platform. Since an important feature of MCDPRs is that the driving cables can only exert a positive pulling force, additional driving cables need to be added to create force containment on the moving platform. The accuracy of the important structural parameters (i.e.,Aim, Biw, $R_{p}$, and $R_{\mathrm{wih}}$) of the kinematic model for MCDPRs directly affects the accuracy of the cable length solution. In practice, errors in structural parameters are inevitable in processes such as robot machining and assembly [38], and the parameters involved in kinematics need to be calibrated for modeling accuracy. Since the pulleys and rollers can be considered as standardized parts, $R_{p}$ and $R_{\mathrm{wih}}$ can be given as design values.

**Figure 6:**
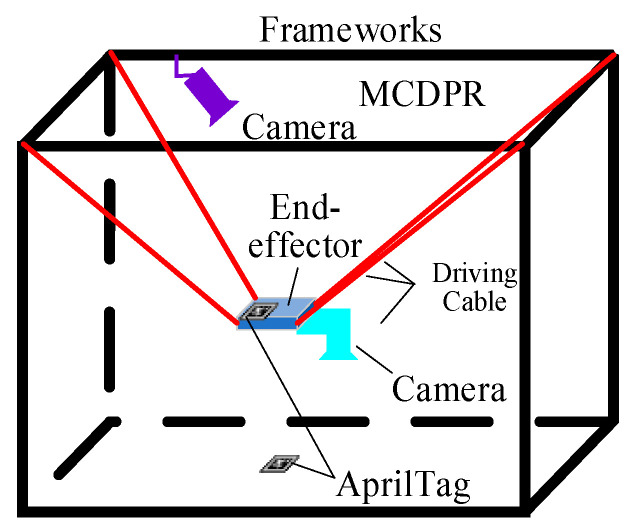
Schematic diagram of a self-calibrating vision system for MCDPRs.

Self-calibration methods usually utilize the sensors that come with the system to obtain observation data [27]; then, they derive theoretical expressions for the observable measurements of the robot based on the structural parameters, compute the deviation between the two, and then construct the objective function, and, finally, iteratively solve for the unknown structural parameters in the kinematics using a Gaussian–Newton method. AprilTag is a type of visual fiducial marker with a unique ID, featuring a simple structure and high robustness to detection [39]. It is commonly used in robotic vision systems for pose estimation and space localization, particularly in tasks involving visual-assisted calibration and identification. In this paper, AprilTag is arranged in the scene to form a self-calibrating vision system with MCDPRs through visually assisted markers, and the scene is shown in Figure 6. The image information acquired by the vision sensor at the tips of the moving platform can be transformed from the world frame to the camera frame, i.e., it satisfies the following:

(17) Hvw=Hmw·Hvm

where Hvw, Hmw, and Hvm are the homogeneous matrices of the AprilTag frame with respect to the world frame, the moving platform frame with respect to the world frame, and the camera frame with respect to the moving platform frame, respectively. According to the positive kinematics model of the MCDPR, Hmw is the pose of the moving platform frame with respect to the world frame, and Hvm is the unknown parameter that needs to be calibrated.

### 4.2. Solving for the Calibration Initial Value

The encoder on the MCDPR winch acquires the cable length and the vision camera acquires the relative position of the camera frame with respect to the AprilTag frame [26,28]. The angle sensor used is an incremental encoder, and the initial angle of rotation of the winch is unknown after the initial machining and assembly are completed, so the initial length of the cable is unknown. Therefore, the length of the *i*th cable, $l_{i}$, can be expressed as follows:

(18) $$l_{i}=\Delta l_{i}+\bar{l_{i}}$$

where $\Delta l_{i}=R_{\mathrm{wih}}\cdot \Delta \theta_{i}$ is the variation in the cable length and $\bar{l_{i}}$ is the original length of the *i*th cable after the assembly of the prototype.

The combination of Equations (18) and (11) can be rewritten as follows:

(19) Fc,iAm,iBw,iPmw,Δli,li¯,Rp=Δli+li¯−fiinvAm,iBw,iPmw,Rp=0

**Figure 7:**
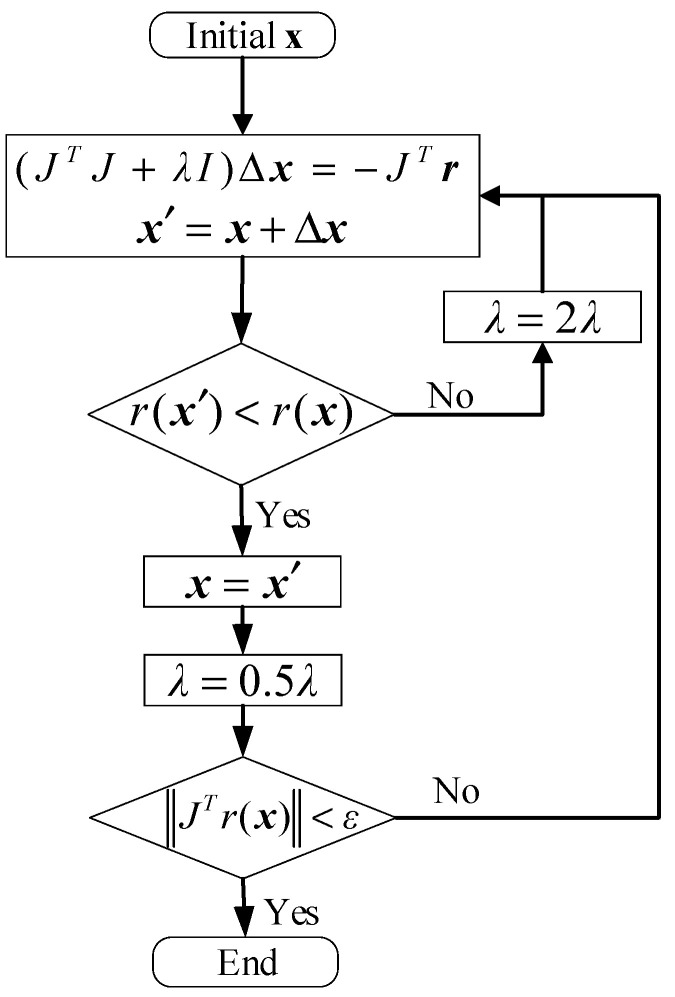
The flowchart of LM algorithm.

Equation (19) gives the relationship between the structural parameter Biw and the sensor measurability, as shown in Figure 7, and the homogeneous matrices of the moving platform frame with respect to the world frame can be written as follows:

(20) Hmw=Htw·Hvt·Hmv

where Htw, Hvt, and Hmv denote the homogeneous matrices of the AprilTag frame with respect to the world frame, the camera frame with respect to the AprilTag frame, and the kinematic coordinate system with respect to the camera frame, respectively.

According to the relationship between the pose and the homogeneous matrices in the Cartesian coordinate system, the pose of the moving platform (i.e., Pmw) with respect to the world frame can be expressed as follows:

(21) xm=Hw1,4mym=Hw2,4mzm=Hw3,4mαm=atan2Hw2,1m,Hw1,1mβm=atan2−Hw3,1m,Hw3,22m+Hw3,32mγm=atan2Hw3,2m,Hw3,3m

Based on Equations (19)–(21), Equation (19) can be further rewritten as follows:

(22) Fc,iAm,iBw,iHw,tHt,vHv,mΔli,li¯,Rp=Δli+li¯−fiinvAm,iBw,iHw,tHt,vHv,mRp

To reduce the dimension of the iterative variables, Equation (22) is solved iteratively by the Gaussian–Newton method; thus, Equation (22) can be rewritten as follows:

(23) Fc,iAm,iBw,iPw,tHt,vPv,mΔli,li¯,Rp=Δli+li¯−fiinvAm,iBw,iPw,tHt,vPv,mRp

The Levenberg–Marquardt (LM) method is suitable for solving this multidimensional optimization problem. However, its convergence efficiency and final solution quality still depend on the choice of the initial values. After the prototype is processed and assembled, the actual values of the structural parameter Biw will not have a large error with the theoretical values, so we propose an initial value solving method for iterative optimization based on this assumption.

Since the initial cable length was difficult to estimate, we eliminated $\bar{l_{i}}$ by differencing multiple sets of calibration data. Assuming that the number of groups of data is *N*, and the number of subsets of data in each group is $n_{c}$, then the size of the calibrated data is $n_{c}N$. Within the subset of the $i\left(i=1,2,\cdots ,N\right)$-th group, note that $f_{i}^{\mathrm{inv}}\left(j\right)$ is the length of the cable obtained by inverse kinematics from the data of the $j\left(j=1,2,\cdots ,n_{c}\right)$-th group, and the difference in Equation (16) is given by the following:

(24) $$\left[\begin{array}{c} F_{c,i}^{\left(j\right)}-F_{c,i}^{\left(j+1\right)}\\ F_{c,i}^{\left(j\right)}-F_{c,i}^{\left(j+2\right)}\\ \vdots \\ F_{c,i}^{\left(j\right)}-F_{c,i}^{\left(j+n_{c}-1\right)}\\ F_{c,i}^{\left(j+1\right)}-F_{c,i}^{\left(j+2\right)}\\ \vdots \\ F_{c,i}^{\left(j+n_{c}-2\right)}-F_{c,i}^{\left(j+n_{c}-1\right)} \end{array}\right]=\left[\begin{array}{c} \Delta l_{i}^{\left(j\right)}-\Delta l_{i}^{\left(j+1\right)}\\ \Delta l_{i}^{\left(j\right)}-\Delta l_{i}^{\left(j+2\right)}\\ \vdots \\ \Delta l_{i}^{\left(j\right)}-\Delta l_{i}^{\left(j+n_{c}-1\right)}\\ \Delta l_{i}^{\left(j+1\right)}-\Delta l_{i}^{\left(j+2\right)}\\ \vdots \\ \Delta l_{i}^{\left(j+n_{c}-2\right)}-\Delta l_{i}^{\left(j+n_{c}-1\right)} \end{array}\right]-\left[\begin{array}{c} f_{i}^{\mathrm{inv}}\left(j\right)-f_{i}^{\mathrm{inv}}\left(j+1\right)\\ f_{i}^{\mathrm{inv}}\left(j\right)-f_{i}^{\mathrm{inv}}\left(j+2\right)\\ \vdots \\ f_{i}^{\mathrm{inv}}\left(j\right)-f_{i}^{\mathrm{inv}}\left(j+n_{c}-1\right)\\ f_{i}^{\mathrm{inv}}\left(j+1\right)-f_{i}^{\mathrm{inv}}\left(j+2\right)\\ \vdots \\ f_{i}^{\mathrm{inv}}\left(j+n_{c}-2\right)-f_{i}^{\mathrm{inv}}\left(j+n_{c}-1\right) \end{array}\right]$$

Equation (24) indicates that $C\left(n_{c},2\right)$ set of equations can be built for any one cable and $C\left(\cdot ,\cdot \right)$ denotes the number of combinations. For a $D_{m}$-DOF robot driven by $N_{r}$ cables, the condition for constructing the super-definite equations is $N_{r}\cdot C\left(n_{c},2\right)\geq n_{c}\cdot D_{m}$. The column vector formed by arranging the left part of Equation (24) in columns is rewritten as follows:

(25) vk=Fc,ijk−Fc,ij+1k⋮Fc,ij+nc−2k−Fc,ij+nc−1k

Thus, the optimization model based on the least square’s method can be written as follows [25,33]:

(26) P1,P2,⋯,PncN=argminP1,P2,⋯,PncN∈Upv1,Tv2,T⋯,vTkT2

where $P_{1},P_{2},\cdots ,P_{n_{c}N}$ denotes the fitted position of the moving platform frame relative to the world frame for each set of calibration data.

Assuming that the pose of the moving platform in each dataset is approximately known, then the approximate fitted value of the homogeneous matrices Hpw is also known. Further, Equation (20) can be rewritten as follows:

(27) Htv·Htw−1=Hmv·Hmw−1

The calibration equation expressed in Equation (27) is similar to the classical “AX=YB” formulation [31,32], which can be quickly solved by the Cronin integral solution method [40]. Let A=Hv,tX=Htw−1,Y=Hv,pB=Hpw−1; Equation (27) can be expressed as follows:

(28) $$R_{A}R_{X}=R_{Y}R_{B}$$

(29) $$R_{A}T_{X}+T_{A}=R_{Y}T_{B}+T_{Y}$$

Vectorizing ***A***, ***X***, ***Y*** and ***B*** results in the following:

(30) $$\begin{array}{c} \mathrm{vec}\left(R_{A}R_{X}\right)=\left(R_{A}⊗I_{3x3}\right)\mathrm{vec}\left(R_{X}\right)\\ \mathrm{vec}\left(R_{Y}R_{B}\right)=\left(I_{3x3}⊗R_{B}^{T}\right)\mathrm{vec}\left(R_{Y}\right) \end{array}$$

where ⊗ denotes the Kronecker product of the matrix, $\mathrm{vec}\left(\cdot \right)$ denotes the column vectorization operation of the matrix, $\mathrm{vec}\left(M\right)={\left[m_{11},m_{12},\cdots ,m_{1s_{2}},\cdots ,m_{s_{1}1},m_{s_{1}2},\cdots ,m_{s_{1}s_{2}}\right]}^{T}$, and M is the operated matrix of $s_{1}\times s_{2}$.

Combining Equations (28) and (30) yields the following:

(31) $$\begin{array}{l} R_{A}R_{X}-R_{Y}R_{B}=\left(R_{A}⊗I_{3x3}\right)\mathrm{vec}\left(R_{X}\right)-\left(I_{3x3}⊗R_{B}^{T}\right)\mathrm{vec}\left(R_{Y}\right)\\ =\left[\begin{array}{cc} \left(R_{A}⊗I_{3x3}\right) & -\left(I_{3x3}⊗R_{B}^{T}\right) \end{array}\right]\left[\begin{array}{c} \mathrm{vec}\left(R_{X}\right)\\ \mathrm{vec}\left(R_{Y}\right) \end{array}\right]=O \end{array}$$

Let $K=\left[\begin{array}{cc} \left(R_{A}⊗I_{3x3}\right) & -\left(I_{3x3}⊗R_{B}^{T}\right) \end{array}\right]$ and $x={\left[\begin{array}{cc} \mathrm{vec}{\left(R_{X}\right)}^{T} & \mathrm{vec}{\left(R_{Y}\right)}^{T} \end{array}\right]}^{T}$. Since the second-order paradigm of the rotation matrix is equal to three, Equation (31) can be expressed as follows:

(32) $$\begin{array}{l} Kx=O\\ s.t.\;\;{\left\|x\right\|}_{2}=\sqrt{6} \end{array}$$

The singular value decomposition (SVD) of matrix K, assuming that $K=U\Sigma V^{T}$, and matrix U is invertible, according to Equation (32), is given by

(33) $$\begin{array}{c} U\Sigma V^{T}x=O\\ \;\;\;\Sigma V^{T}x=O \end{array}$$

Let $\zeta =V^{T}x$. The following objective optimization equation can be established according to Equation (33):

(34) $$\underset{x}{\min}\;\xi^{T}\Sigma^{T}\Sigma \xi =\underset{x}{\min}\;x^{T}V\Sigma^{T}\Sigma V^{T}x$$

where Σ denotes that the singular values $\sigma_{i}$ of the matrix K are distributed from largest to smallest on the diagonal of the matrix, Σ=σ1    σ2    ⋱    σn, and $\sigma_{i}=\sqrt{\lambda_{i}}$, $\lambda_{i}$ is the eigenvalue of $K^{T}K$.

Thus, Equation (34) can be further rewritten as follows:

(35) minx ξTΣTΣξ=minx ξTλ1    λ2    ⋱    λnξ

Since $\sigma_{1}>\sigma_{2}>\cdots >\sigma_{n}$, the objective function expressed in Equation (35) is minimized when $\xi ={\left[0,0,\cdots ,\sqrt{6}\right]}^{T}$, i.e.,

(36) $$x=V\zeta =\sqrt{6}v_{n}$$

where $v_{n}$ denotes the eigenvector corresponding to the smallest eigenvalue of the matrix $K^{T}K$.

From Equation (36), the rotational components of the unknown quantities X and Y can be solved, and for the translational vectors, Equation (29) is deformed; then,

(37) $$R_{A}T_{X}-T_{Y}=R_{Y}T_{B}-T_{A}$$

Similarly, one obtains the following:

(38) $$\begin{array}{cc} {}[R_{A} & -I_{3x3}] \end{array}\left[\begin{array}{c} T_{X}\\ T_{Y} \end{array}\right]=R_{Y}T_{B}-T_{A}$$

Let $Q=\begin{array}{cc} {}[R_{A} & -I_{3x3}] \end{array}$, $x={\left[\begin{array}{cc} T_{X}^{T} & T_{Y}^{T} \end{array}\right]}^{T}$, and $b=R_{Y}T_{B}-T_{A}$. Then, Equation (38) is solved by the Moore–Penrose method, which yields the following:

(39) $$\begin{array}{c} Qx=b\\ x={\left(Q^{T}Q\right)}^{-1}Q^{T}b \end{array}$$

Combining Equation (36) with Equation (39), the rotational and translational components of XHtw−1,YHpv can be solved, and the corresponding homogeneous matrices can be determined. Based on the transformation relationship between the pose and the homogeneous matrices in the Cartesian coordinate system, Ptw and Pmv can be further solved, which in turn determines the iterative unknown initial values of the calibration.

### 4.3. Establishment and Resolving of Self-Calibrating Equations

Let x=Bw,1,xBw,1,yBw,1,z⋯,Bw,4,xBw,4,yBw,4,zl1¯,l2¯,l3¯,l4¯,Pw,tTPmTvT. Expressing the cable length model in Equation (23) as a function of x gives the following:

(40) $$F_{c}\left(x\right)=\left[\begin{array}{c} F_{c,1}\left(x\right)\\ F_{c,2}\left(x\right)\\ F_{c,3}\left(x\right)\\ F_{c,3}\left(x\right) \end{array}\right]$$

Due to the coupling between the unknown quantities,

Pw,tTPmTv and Bw, and the large dimensionality of the variables, the cable length model as the objective function simply by minimizing Equation (40) is prone to fall into a local optimum that is far away from the true values of the variables. Considering that the difference between the true value of the fixed anchor seat and the theoretical value is small when the robot is processed and assembled, we introduce a soft constraint to limit the range of values of the fixed anchor seat in the variables, and the soft constraint is as follows:

(41) Fb,ix=Biw−Bi′w2

(42) $$F_{b}\left(x\right)=\left[\begin{array}{c} F_{b,1}\left(x\right)\\ F_{b,2}\left(x\right)\\ F_{b,3}\left(x\right)\\ F_{b,3}\left(x\right) \end{array}\right]$$

where Bi′w denotes the theoretical coordinate value of the *i*th fixed anchor seat.

Assembling Equation (40) by $n_{c}N$ sets of calibrated data yields the following:

(43) Mcx=Fc1xFc2x⋮FcncNx

Thus, we can build the following optimization model:

(44) $$x=\underset{x}{\arg\;\min\;}\lambda_{1}{\left\|M_{c}\left(x\right)\right\|}_{2}+\lambda_{2}{\left\|F_{b}\left(x\right)\right\|}_{2}$$

Similarly, Equation (44) can be solved iteratively by the LM algorithm. Parameters $\lambda_{1}$ and $\lambda_{2}$ are associated with the algorithm; in this study, we set $\lambda_{1}$ = 5 and $\lambda_{2}$ = 1. The flowchart of the LM algorithm is shown in Figure 7, where $r(x)=\left[\begin{array}{c} \sqrt{\lambda_{1}}M_{c}(x)\\ \sqrt{\lambda_{2}}F_{b}(x) \end{array}\right]$, $J=\frac{\partial r(x)}{\partial x}$, λ represents the Damping factor, and ε denotes the gradient tolerance.

## 5. Vision Guidance-Based Bin-Picking Method for MCDPRs

Based on the calibrated structural parameters, this section presents the vision-guided bin-picking method for the MCDPR. In the present system, the visual pipeline consists of grasping-frame detection using AFFGA-Net [41] on RGB images, depth recovery from the aligned depth map, and coordinate transformation from the camera frame to the world frame. After the grasping frame is determined in the image coordinate system, the corresponding grasp point is mapped into the robot reference coordinate system through kinematic transformation, and the robot then executes the corresponding grasping motion.

**Figure 8:**
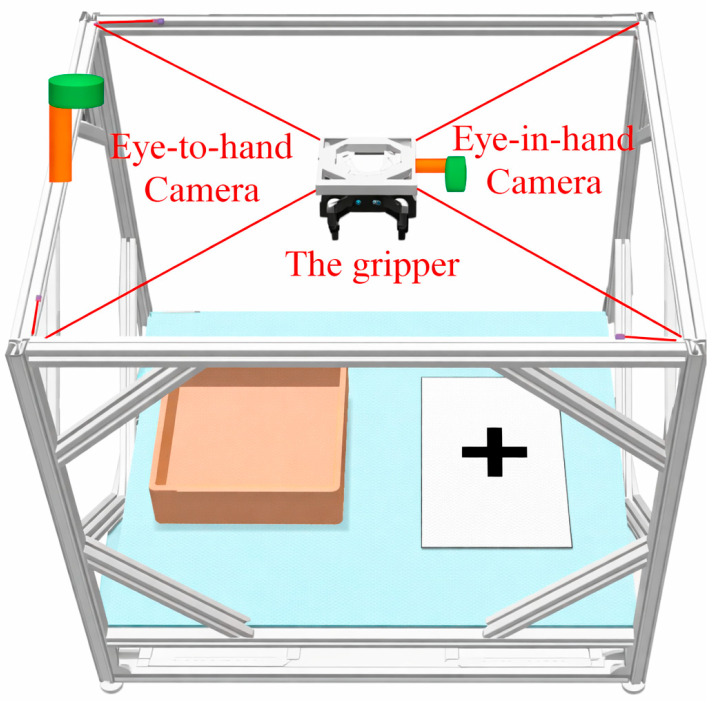
Schematic diagram of a MCDPR for bin-picking.

**Figure 9:**
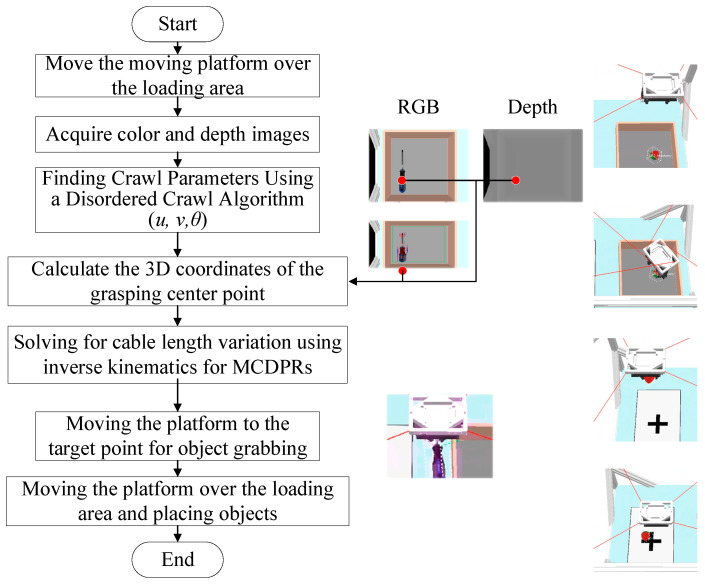
The flowchart of the pickup method for MCDPRs.

As shown in Figure 8, a schematic diagram of vision-guided unordered grasping for an MCDPR is shown in Figure 9, in which the orange area places the object to be picked up and the white cross area unloads the grasped object.

If the coordinates of the target grasping point in the pixel coordinate system of the image are $p_{uv}={\left[u,v,1\right]}^{T}$, then the pose relationship between the target grasping point coordinates in the pixel coordinate system and the world coordinate system can be described as follows:

(45) zopuv=Kv·Rext·pow+Text

where $z_{o}$ denotes the depth of the target grasping point in the camera frame and pow denotes the 3D position of the target grasping point relative to the world frame.

The $z_{0}$ in Equation (45) can be obtained from the depth map, which needs to be aligned due to the inconsistency of the imaging coordinate system of the color image and the depth image in the color-depth camera [42]. At this point, the color camera and the depth camera are equivalent to binocular cameras, and the mapping relationship can be expressed as follows:

(46) $$p_{uv}^{l}=W_{r2l}\cdot p_{uv}^{r}$$

where $p_{uv}^{l}$ denotes the homogeneous coordinates of the left-eye camera pixels, $W_{r2l}$ is the mapping matrix from the right-eye camera to the left-eye camera, and $p_{uv}^{r}$ denotes the homogeneous coordinates of the right-eye camera pixels.

According to the self-calibration method, the relative relationship Hvm between the moving platform coordinate system and the camera coordinate system is known, then the external reference matrix of the camera can be expressed as follows:

(47) Hext=Hvw=Hmw·Hvm

Let $R_{\mathrm{ext}}$ and $t_{\mathrm{ext}}$ be the rotation matrix and translation vector of $H_{\mathrm{ext}}$, respectively. According to Equation (45), the coordinates of the target grasping point relative to the world coordinate system can be expressed as follows:

(48) pow=1zoRext−1·Kv−1·puv−Rext−1·text

Since the tips of the moving platform carries the gripper and is rigidly connected, the center of the end-effector (also known as Tool Center Point (TCP)) is the grasping center, so the center of the end-effector can be determined directly from the theoretical values of the design parameters. Assuming that the three-dimensional position of the TCP with respect to the moving platform is ptm, according to Equation (48), the desired position (i.e., pmw) of the moving platform can be characterized as follows:

(49) pmw=pow−ptm

## 6. Simulation and Experiment

This section validates the proposed design and methods from three aspects: model-related accuracy, calibration effectiveness, and task-level feasibility. The simulation results are first used to verify the correctness of the proposed modeling and calibration framework under controllable conditions, and the hardware experiments are then used to evaluate the calibration improvement, trajectory-tracking performance, grasping feasibility, and anti-disturbance tension-maintenance capability of the prototype.

### 6.1. Simulation

**Figure 10:**
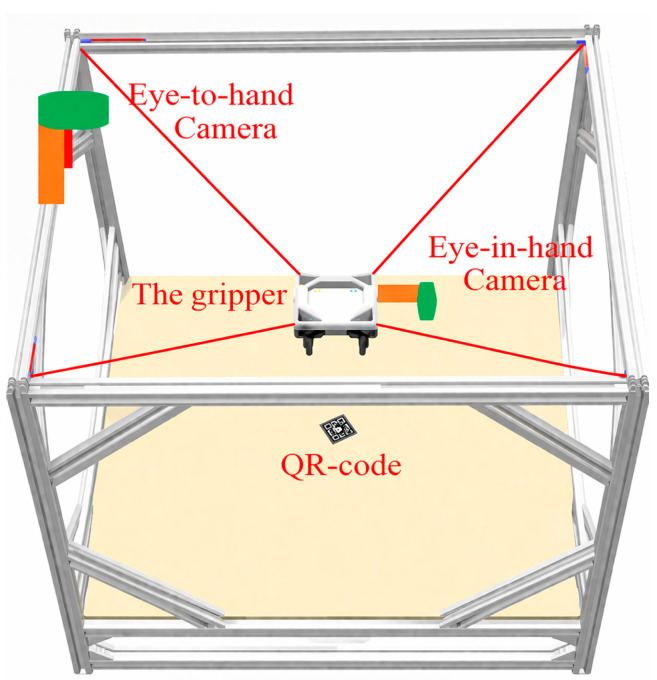
Prototype simulation model in CoppeliaSim.

In order to verify the kinematics, self-calibration, and grasping method, a simulation model was built in the CoppeliaSim (version 4.4.0) simulation environment, as shown in Figure 10.

Since CoppeliaSim can only model robots using general joints such as translation, rotation, and spherical joints, there are primarily two methods for simulating cables. The first method, called the SPS structure, connects rigid bodies using translational joints and simulates the connection between the anchor point and the moving platform using spherical joints. The change in cable length is achieved by adjusting the angles of the translational joints. The second method involves rendering representation, where the position relationship between the fixed anchor points and the moving platform is first calculated using an API, and then the simulated cable connection is drawn based on this information. The first method requires calculating the angles of multiple translational joints to determine cable length, making it suitable for models with small anchor holes but difficult to apply to complex pulley systems. In contrast, the second method directly calculates the actual cable length using the API, making it simpler and more adaptable. Therefore, the second method was chosen for cable simulation in this study. An additional RGB-D camera and gripper were installed outside the moving platform in order to realize bin-picking based on vision, which obtained the visual information of the scene and realized the object pickup.

#### 6.1.1. Numerical Simulation of Self-Calibration Method

**Figure 11:**
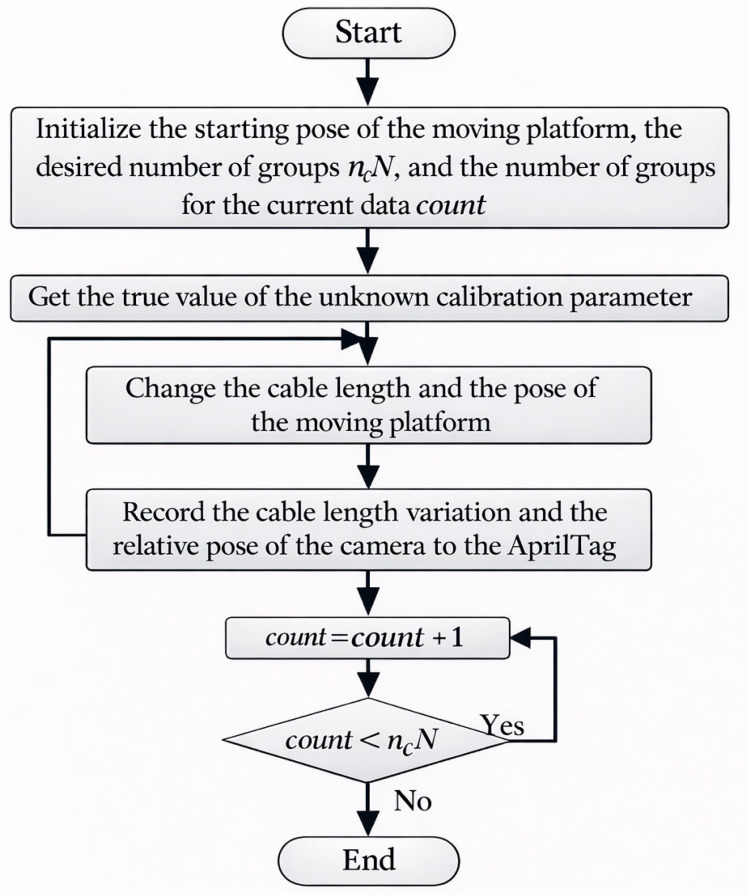
The flowchart of calibration data processing.

Considering the structural parameter errors caused by the actual part processing and prototype assembly, we added random errors of mean of 0 and an amplitude of 0.005 to the fixed anchor point unit. The pose of the moving platform was changed many times by CoppeliaSim’s Application Programming Interface (API), and the sensor data under each group of poses was recorded (i.e., the variation in cable length and the pose measured by the AprilTag). After obtaining enough data, the truth value to be calibrated of the unknown variables, x=Bw,1,xBw,1,yBw,1,z⋯,Bw,4,xBw,4,yBw,4,zl1¯,l2¯,l3¯,l4¯,Pw,tTPmTv, is obtained through the API. The detailed flowchart for obtaining calibration data is shown in Figure 11.

**Figure 12:**
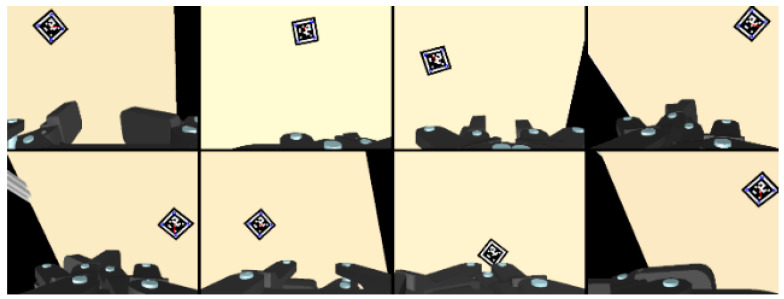
The pose variation in the moving platform.

Figure 12 shows representative intermediate poses collected during the calibration process, and the corresponding numerical results are listed in Table 3. The calibrated structural parameters are close to the ground-truth values generated in simulation. Quantitatively, the position error is within 0.05 mm, the attitude error is within 0.02°, and the average cable length error is 0.009 mm. These results indicate that, under noise-controlled simulation conditions, the proposed self-calibration method can accurately recover the structural parameters and the camera platform relative pose. This also supports the validity of the proposed kinematic model, because the calibration process is built directly on the consistency between the measured cable lengths, visual observations, and the modeled geometric relationships.

#### 6.1.2. Simulation of Bin-Picking Based on Visual Guidance

**Figure 13:**
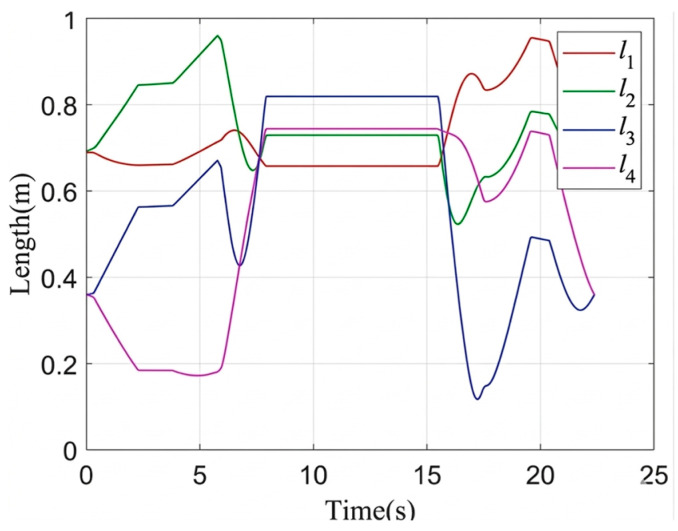
The variation in the cable length during operation.

**Figure 14:**
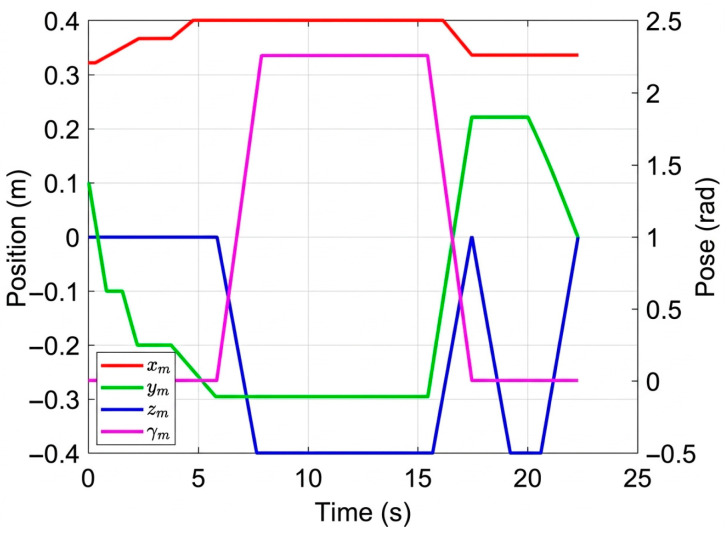
The pose variation in the moving platform during operation.

**Figure 15:**
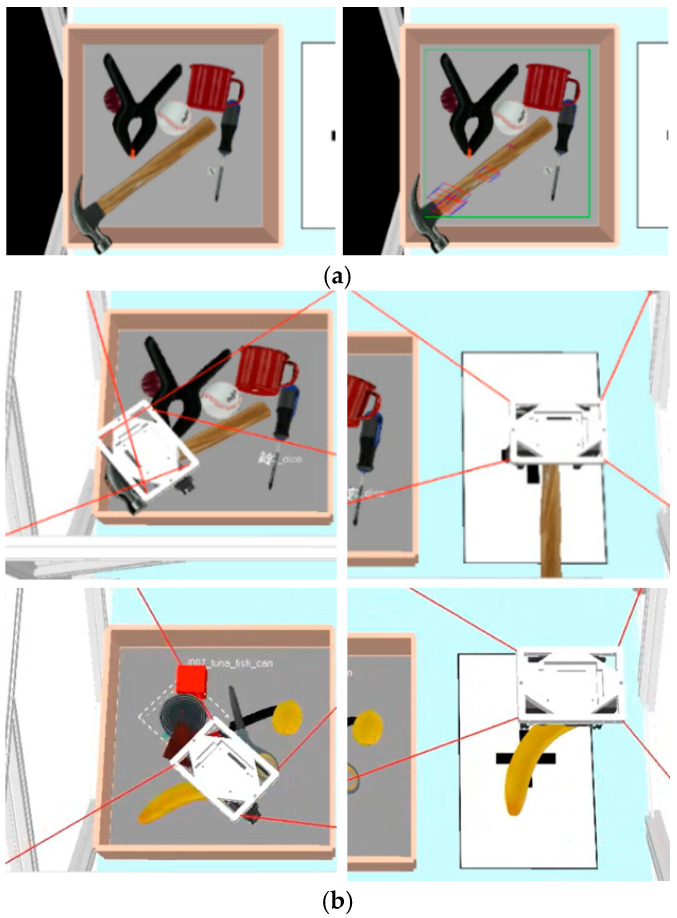
Visual-based bin-picking process. (**a**) Grasping frame detected by AFFGA-net [41]. (**b**) Expected pose of the moving platform.

The color camera is used as the left camera, while the depth camera is used as the right camera. The above method was verified in the YCB dataset proposed by Yale University [43]. The simulation of the MCDPR grasping process is shown in Figure 13, Figure 14 and Figure 15.

The simulation results in Figure 13, Figure 14 and Figure 15 illustrate the complete grasping process, including grasp detection, pose conversion, and motion execution. To evaluate the contribution of self-calibration to grasping accuracy, two groups of structural parameters were used in simulation: the nominal design values and the calibrated values. The corresponding end poses are listed in Table 4. After self-calibration, the Euclidean position error decreases from 0.371 mm to 0.048 mm, indicating a significant improvement in positioning accuracy, while the grasping orientation error decreases from 0.071° to 0.004°. This comparison shows that the proposed grasping pipeline is not only dependent on visual detection, but also strongly benefits from the improved geometric consistency provided by the calibration stage. Therefore, the simulation results show that grasping performance benefits from the complete pipeline of modeling, calibration, and motion execution.

### 6.2. Experiment

The hardware experiments are organized into four aspects: preliminary actuator/sensor calibration, self-calibration of structural parameters, task-level motion and grasping execution, and redundant cable tension maintenance under disturbance. This organization reflects the validation logic from hardware preparation to model correction, task execution, and disturbance response.

#### 6.2.1. Preliminary Actuator and Sensor Calibration

Before evaluating the robot-level calibration and task execution, the motor deceleration ratio and the tension sensor were calibrated to reduce hardware-level measurement uncertainty. These two procedures provide the basic actuator and sensing accuracy required for the subsequent self-calibration and motion experiments.

Due to the error between the actual and theoretical reduction ratio of the motor reducer, it is necessary to re-calibrate the motor reduction ratio. The relationship between motor reduction ratio and driver pulse value can be expressed as follows:

(50) $$\rho =\frac{N_{\mathrm{motor}}}{4n_{\mathrm{enc}}}$$

Since the external encoder is directly connected to the cable roller, the angle of the roller rotation is equal to the angle of the encoder shaft rotation, which means the relationship between the expected cable length change and the servo motor pulse can be measured through the encoder data. In the experiment, the driver is connected to the motor, and the phases A and B of the external encoder are connected to the timer multiplexing channel of the STM32 microcontroller (STM32F103C8T6, ALIENTEK, Guangzhou, China). The motor reset command is sent through the bus of the STM32 microcontroller, and the motor enters the position control mode. At the same time, we set the timer count to 0 and the upper limit of the count to 4095. The upper computer successively sends position instructions to the STM32 microcontroller to make the motor move. When the timer count overflows and interrupts, the upper computer stops sending instructions, records the position difference during one rotation of the drum, and then gets the relationship between the position of the motor and the rotation angle of the drum. The average deceleration ratio of the motor is 371.13 after several experiments.

After calibrating the motor-side transmission relationship, the cable tension measurement was further calibrated. During the experiment, due to temperature changes and unstable power supply voltage, the tension sensor has zero drift and its linear ratio has certain errors. The guiding pulley of the MCDPR exerts pressure on the resistance strain gauges on the tension sensor at a different angle after the cable is wrapped around it, which makes the actual range different from the standard range, and therefore needs to be calibrated.

The range of the single pulley tension sensor is 0~50 N, and the signal after the output of the transmitter is 0~3.3 V, so the theoretical linear ratio $k_{\exp}^{t}$ of the transmitter output to the cable tension is $50/3.3\approx 15.15\left(N/V\right)$, and the theoretical tension is as follows:

(51) $$y_{\exp}^{t}=k_{\exp}^{t}\cdot u_{r}$$

where $y_{\exp}^{t}$ is the theoretical pull force and $u_{r}$ is the actual output voltage of the transmitter.

The driving cable is led through the drum, around the measuring wheel of the tension transducer, through the rotating pulley at the fixed anchor point seat, and then connected to the naturally sagging weights, with the cable end using a double clevis to hold the weights in place. If the mass of the cable and the double clamp chuck is ignored, the calibration model of the tension sensor can be expressed as follows:

(52) $$\begin{array}{l} y_{r}^{t}=k_{r}^{t}\cdot \left(u_{r}-u_{0}\right)\\ y_{\max}^{t}=k_{r}^{t}\cdot \left(u_{\max}-u_{0}\right) \end{array}$$

where $y_{r}^{t}$ is the actual tension value, $k_{r}^{t}$ is the actual linear ratio of the sensor, $u_{0}$ is the zero voltage, $y_{\max}^{t}$ is the maximum measurable cable tension of the tension sensor, and $u_{\max}=3.3$ V.

**Figure 16:**
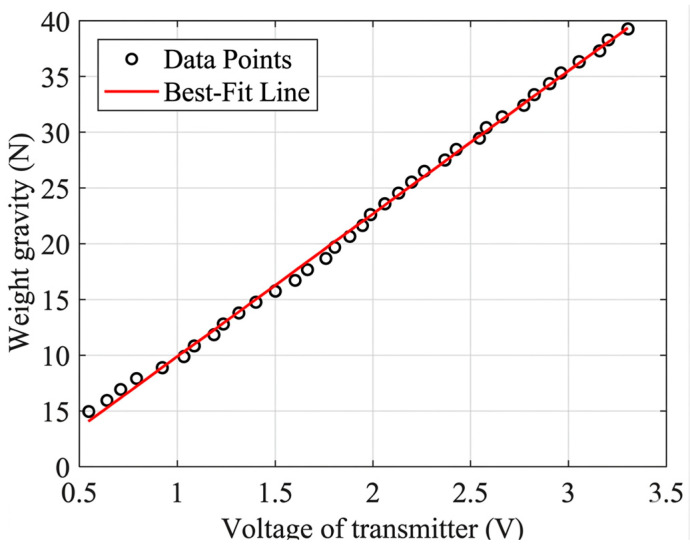
Calibration results for tension sensors.

At the ends of the cable, a 500 g hanging disk is attached, and different weight combinations are used to get readings of the transmitter’s output under different loads. According to Equation (52), the calibration results are shown in Figure 16, where $k_{r}^{t}=12.77\left(N/V\right)$, $u_{0}=0.2355\left(V\right)$, and $y_{\max}^{t}=39.1434\left(N\right)$.

#### 6.2.2. Self-Calibration Performance of Structural Parameters

**Figure 17:**
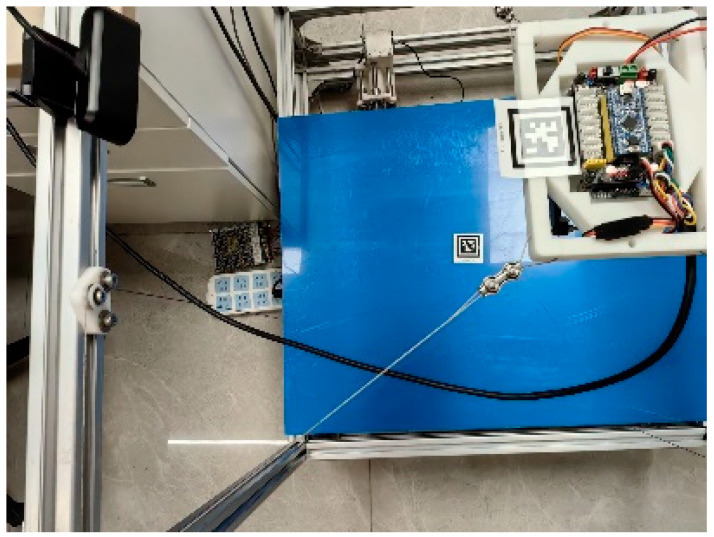
Experimental scenario diagram.

**Figure 18:**
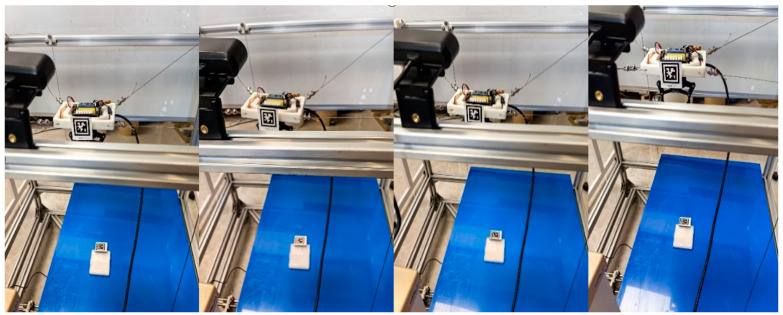
Schematic representation of the self-calibration process of MCDPRs.

As shown in Figure 17, This experiment evaluates the practical effect of the proposed self-calibration method on the assembled prototype. As shown in Figure 18, The moving platform was driven to multiple poses, while the cable length data from the encoders and the AprilTag pose measurements from the onboard camera were recorded simultaneously. Based on 20 valid datasets, the structural parameters were identified, and the results are listed in Table 5. To further evaluate the practical benefit of calibration, an external global camera was used to observe the moving platform at the start point and end point, and the measured poses before and after calibration are summarized in Table 6. The relative position error is reduced by 58.33% after calibration, indicating improved geometric consistency of the assembled prototype under the available experimental conditions.

A comparison with the simulation results further supports the effectiveness of the proposed self-calibration method. In simulation, the Euclidean position error decreases from 0.371 mm to 0.048 mm after calibration, while the orientation error decreases from 0.071° to 0.004°. In the hardware experiments, the relative position error is reduced by 58.33% after calibration. These results consistently show that self-calibration improves the geometric consistency and positioning accuracy of the MCDPR in both simulation and prototype experiments.

#### 6.2.3. Trajectory Execution and Grasping Experiments

This group of experiments evaluates whether the calibrated model can support task-level motion execution on the hardware prototype. The experiments include circular trajectory tracking and two representative grasping-related motion tasks. Together, they examine the consistency between model-based cable length commands, realized platform motion, and grasp-related transport behavior.

Circular trajectory tracking was first conducted to evaluate continuous task-space motion based on the calibrated kinematic model. To make the motion trajectory smoother, the cubic spline interpolation method is utilized to interpolate the individual segmented trajectories, and the cable length variation velocity at any moment t can be expressed as follows:

(53) $$\begin{array}{l} S_{i}^{\left(k\right)}\left(t\right)=s_{i,0}^{\left(k\right)}+s_{i,1}^{\left(k\right)}\left(t-t_{0}^{\left(k\right)}\right)+s_{i,2}^{\left(k\right)}{\left(t-t_{0}^{\left(k\right)}\right)}^{2}+s_{i,3}^{\left(k\right)}{\left(t-t_{0}^{\left(k\right)}\right)}^{3}\;\mathrm{if}\;t_{0}^{\left(k\right)}\leq t<t_{0}^{\left(k+1\right)}\\ V_{i}^{\left(k\right)}\left(t\right)=s_{i,1}^{\left(k\right)}+2s_{i,2}^{\left(k\right)}\left(t-t_{0}^{\left(k\right)}\right)+3s_{i,3}^{\left(k\right)}{\left(t-t_{0}^{\left(k\right)}\right)}^{2}\;\mathrm{if}\;t_{0}^{\left(k\right)}\leq t<t_{0}^{\left(k+1\right)} \end{array}$$

where $s_{i,0}^{\left(k\right)},s_{i,1}^{\left(k\right)},s_{i,2}^{\left(k\right)},s_{i,3}^{\left(k\right)}$ is the 3th-degree polynomial coefficient of the *k*th trajectory of the *i*th cable, *k* is the number of interpolation points, and $t_{0}^{\left(k\right)}$ is the moment at the beginning of the *k*th trajectory.

Here, the control part of the motor performs the position control mode and the remaining motor performs the current control mode, so that the cable length and speed can be mapped to the pulse position value and speed of the motor:

(54) $$\begin{array}{c} q_{i}^{\mathrm{motor}}=\frac{\rho \left(l_{i}^{\exp}-l_{i}^{\mathrm{real}}\right)}{2\pi R_{w}}\\ v_{i}^{\mathrm{motor}}=\frac{\rho v_{i}^{\exp}}{2\pi R_{w}} \end{array}$$

where $l_{i}^{\exp},l_{i}^{\mathrm{real}}$ denote the desired and actual lengths of the *i*th cable at time t, respectively, $l_{i}^{\exp}=S_{i}^{\left(k\right)}\left(t\right)$, $q_{i}^{\mathrm{motor}},v_{i}^{\mathrm{motor}}$ denote the desired pulse position and the desired rotational velocity of the *i*th motor, respectively, and $v_{i}^{\exp}=V_{i}^{\left(k\right)}\left(t\right)$.

**Figure 19:**
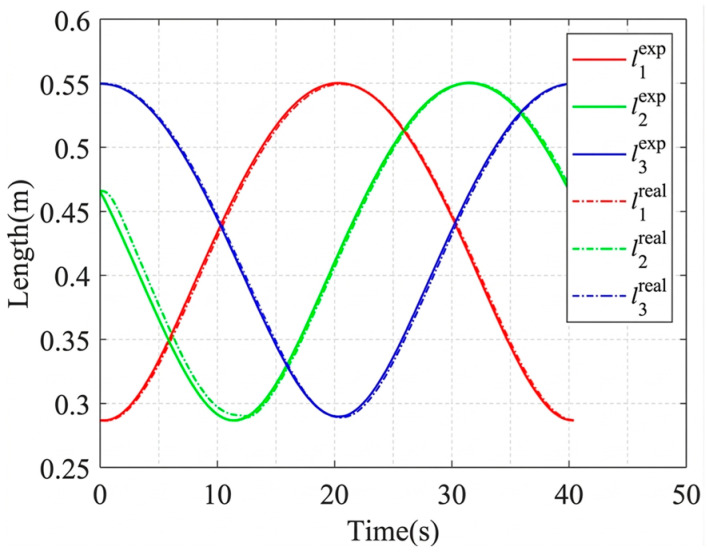
The variation in the cable length.

**Figure 20:**
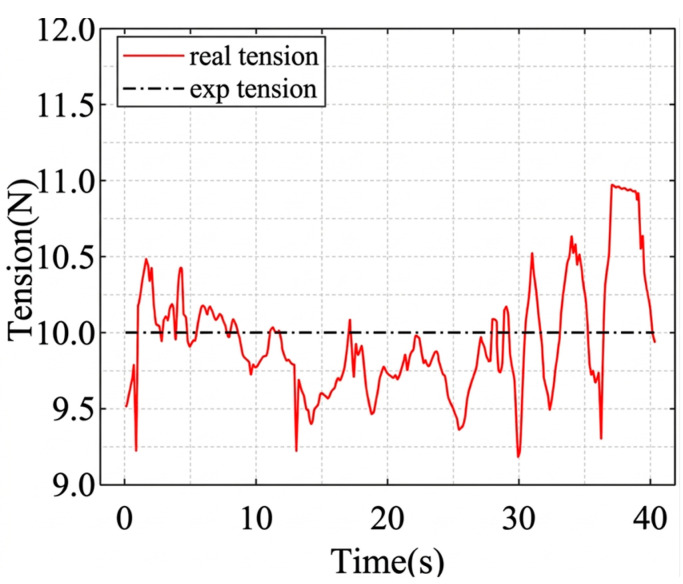
The variation in the cable tension.

**Figure 21:**
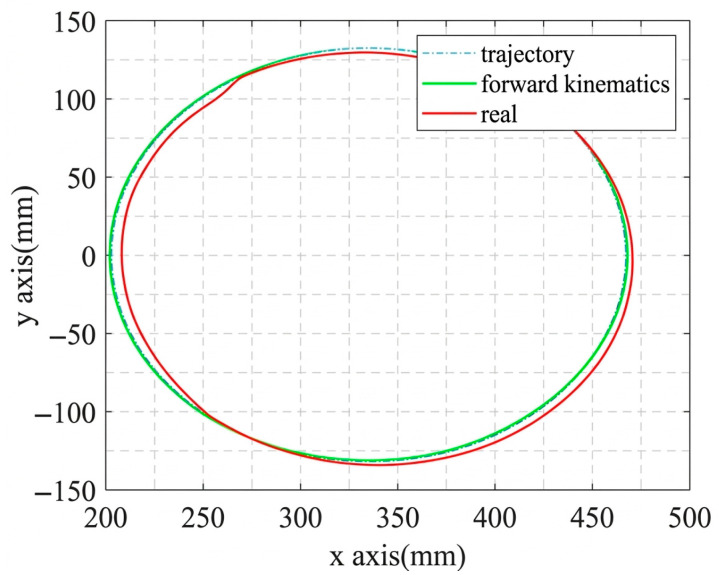
Comparison of the trajectory tracking results for the moving platform.

**Figure 22:**
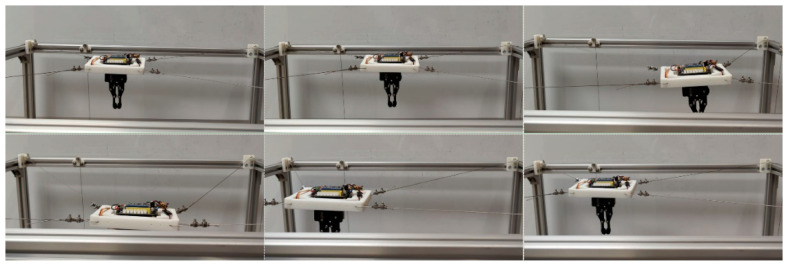
The circular trajectory tracking results for the MCDPR.

The circular trajectory parameters are given in Table 7, and the corresponding cable length, cable tension, and platform pose results are shown in Figure 19, Figure 20, Figure 21 and Figure 22. As shown in Figure 21, the desired pose and the actual pose remain generally consistent along the commanded circular path. Quantitatively, the cable length error remains small overall, with an average value of 1.24 mm, a standard deviation of 1.062 mm, and a maximum value of 4.105 mm, while the minimum error is close to zero at some time instants when the desired and actual cable lengths nearly overlap. The trajectory error is 3.4251 mm on average and reaches 7.0437 mm at the peak. In addition, the cable tension stays close to the desired value throughout most of the motion, and its mean absolute error is about 0.291 N, indicating relatively stable tension regulation during circular tracking. The relatively larger error at the beginning of motion, especially for cable 2, is mainly attributed to the motor acceleration delay, which introduces transient hysteresis between the desired and actual cable lengths.

These results suggest that the model-based mapping from platform motion to cable length command is able to support circular trajectory execution on the prototype. The remaining error is influenced not only by geometric modeling accuracy but also by actuation dynamics, especially during the initial acceleration stage.

**Figure 23:**
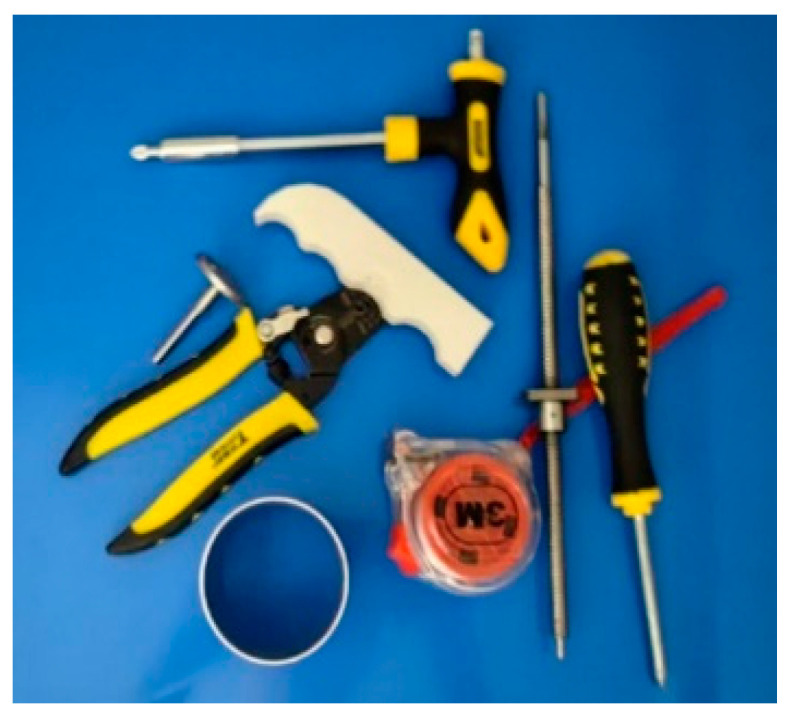
Schematic diagram of grasping randomly placed objects.

**Figure 24:**
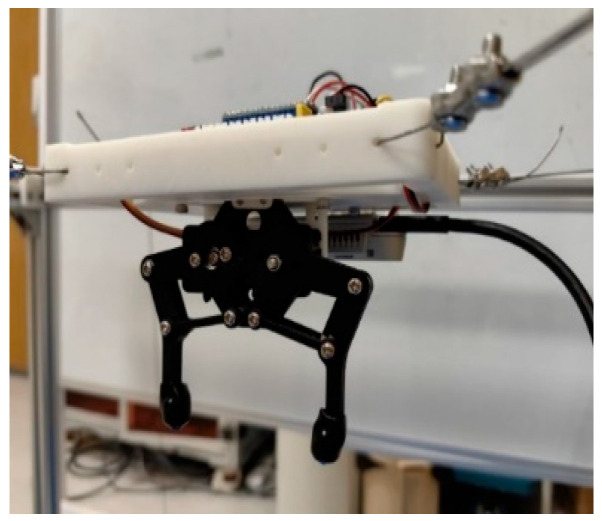
Initial state diagram of the moving platform.

**Figure 25:**
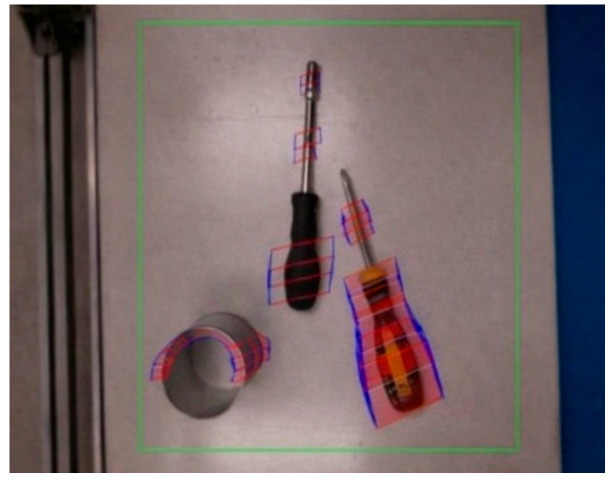
Schematic diagram of capture frame detection.

**Figure 26:**
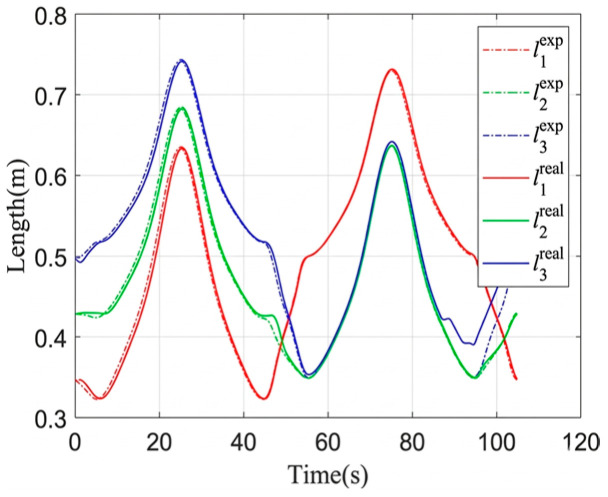
The variation curve of the cable length.

**Figure 27:**
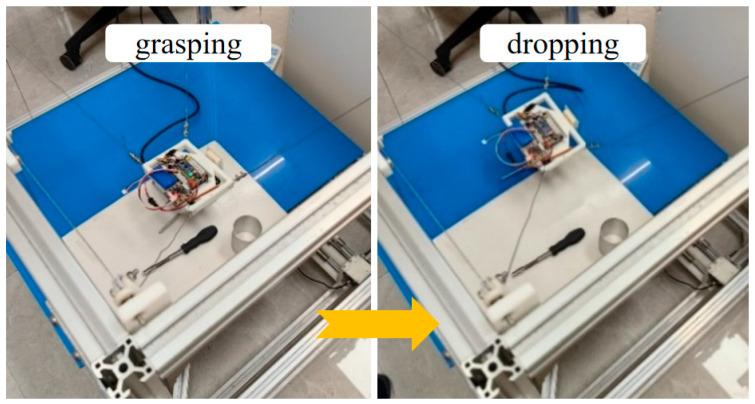
Schematic diagram of the grasping and dropping process.

A representative grasping case was then used to evaluate the complete vision-guided grasping pipeline on randomly placed objects. The purpose of this experiment is to verify the end-to-end feasibility of the proposed method, including visual perception, grasp-point detection, coordinate transformation, and robot execution of grasping, transfer, and release, rather than to focus on detailed motion-consistency analysis during transport. In this experiment, the schematic diagram of randomly placed objects to be grasped is shown in Figure 23, including screwdrivers, 3D-printed parts, CNC-machined parts, etc. The initial state of the mobile platform equipped with the gripper and the Intel Realsense D415 camera (Intel RealSense D415, Intel Corporation, Santa Clara, CA, USA) is shown in Figure 24. The procedure includes scene initialization, grasp-point estimation from RGB-D images, and robot execution of the grasping motion. Specifically, the Intel RealSense D415 camera acquires aligned RGB-D data, and the RGB image is processed by AFFGA-Net [41] to detect the grasping frame. The corresponding target position in the world frame is then obtained through the coordinate transformation model introduced in Section 5. In the representative case shown in Figure 25, the first predicted grasp point in the pixel frame is (360, 285), and the converted 3D target position in the world frame is [252, −39, −517] mm. Based on this target, the desired cable lengths are computed from the calibrated kinematic model, and the moving platform is driven to complete grasping, object transfer, and object release. The release position is [335, 100, −510] mm, after which the platform returns to [335, 0, −9] mm. The cable length variation during the process is shown in Figure 26, while Figure 27 illustrates the grasping and releasing states. These results show that the detected grasp point can be transformed into executable robot motion for grasping, transfer, and release on the prototype.

**Figure 28:**
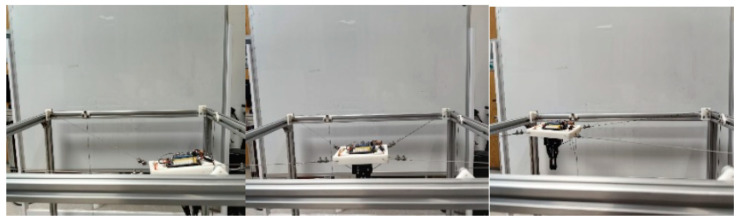
The trajectory diagram of the MCDPR gripping a screw.

**Figure 29:**
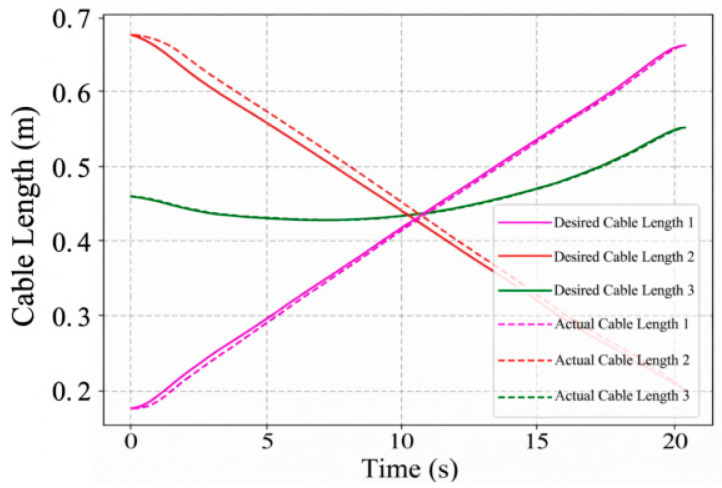
Cable length variation curve for linear trajectory.

**Figure 30:**
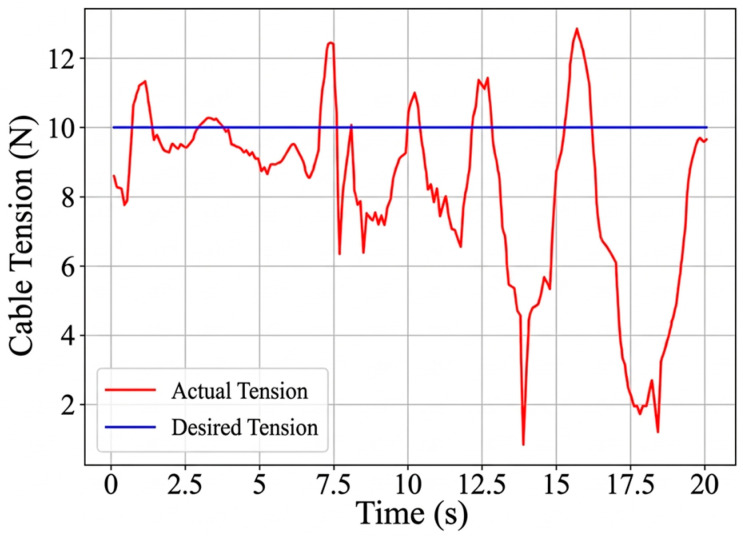
Cable tension variation curve for cable 4 along the linear trajectory.

To complement the above task-level grasping demonstration, a second grasping-related motion case was further considered to evaluate the cable-response consistency during linear transfer. Unlike the previous experiment, which emphasizes the feasibility of the complete vision-guided grasping pipeline, this experiment focuses on the execution behavior during the post-grasp transport stage. The corresponding robot state and cable responses are shown in Figure 28, Figure 29 and Figure 30, and the cable length errors are summarized in Table 8. The average cable length variation error is 1.13 mm, with a standard deviation of 0.82 mm. This error level is on the same millimeter scale as that observed in the circular trajectory experiment, indicating that the proposed model and control framework can maintain stable execution accuracy during grasp-related transport on the hardware prototype. Figure 30 shows that the redundant cable tension exhibits dynamic variation around the desired value during transport, while the cable length error remains small. This suggests that the tension response is mainly associated with the continuous adjustment of the redundant cable during motion, and that the overall transport process remains stable.

#### 6.2.4. Disturbance Response of Redundant Cable Tension

**Figure 31:**
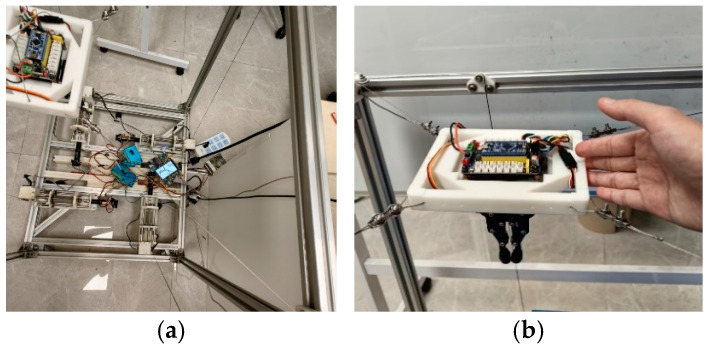
The moving platform. (**a**) Constant tension experiment with the moving platform stationary. (**b**) Constant tension experiment with the moving platform under disturbance.

**Figure 32:**
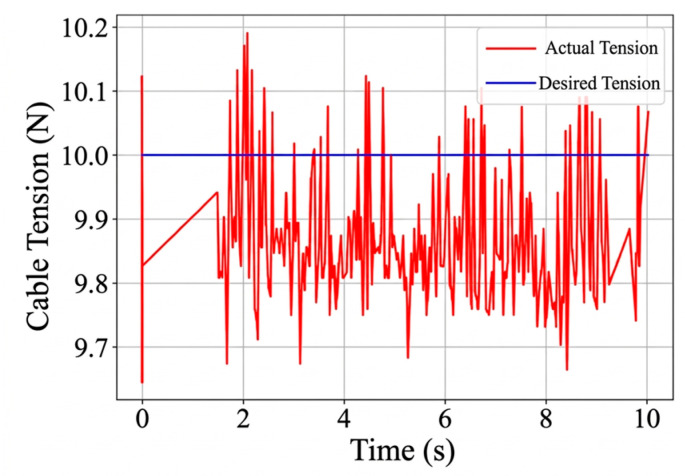
Cable tension control curve under static conditions.

**Figure 33:**
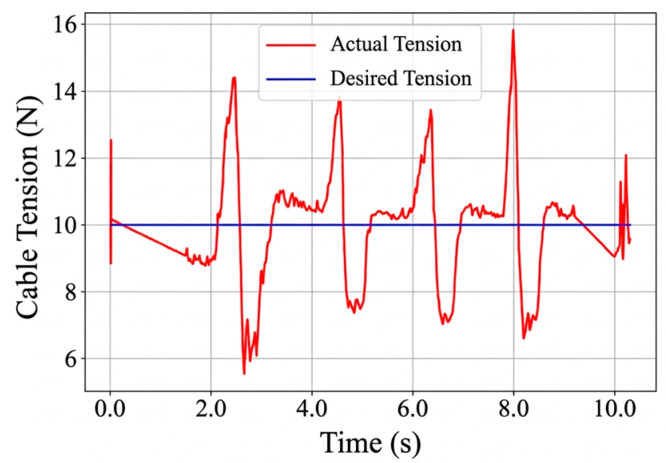
Cable tension control curve under disturbed conditions.

This experiment evaluates the disturbance-response behavior of the redundant cable tension-maintenance strategy. The corresponding results are shown in Figure 31, Figure 32 and Figure 33. Under static conditions, the steady-state tension error is approximately 0.2 N relative to the desired value. After an external disturbance, the cable tension returns toward the desired range within about 1 s. During the test, the redundant cable tension remains within 10 N ± 0.5 N, indicating that the controller can restore the target tension range within a short settling period.

## 7. Conclusions

This paper presented a modular cable-driven parallel robot (MCDPR) together with its kinematic modeling, vision-based self-calibration, and vision-guided grasping methods. A compact modular architecture integrating drive, sensing, and cable-guiding functions was developed, and the corresponding modeling, calibration, and grasping strategies were established and validated. Specifically, a pulley-considered multilayer kinematic model was established, and a vision-based self-calibration method together with a vision-guided bin-picking framework was further developed.

The proposed methods were validated by both simulation and hardware experiments. In simulation, self-calibration reduced the Euclidean grasping position error from 0.371 mm to 0.048 mm and the orientation error from 0.071° to 0.004°. In hardware experiments, the relative position error was reduced by 58.33% after self-calibration. In circular trajectory tracking, the average and maximum cable length errors were 1.24 mm and 4.105 mm, respectively. In the disturbance experiment, the redundant cable tension was maintained within 10 N ± 0.5 N and recovered within about 1 s after disturbance. These results demonstrate that the proposed modular prototype and associated methods can support calibration, trajectory execution, tension maintenance, and vision-guided grasping on the experimental platform.

Overall, the present study combines modular mechanical design, pulley-considered kinematic modeling, vision-based self-calibration, and vision-guided grasping within a unified MCDPR framework. The results from both simulation and hardware experiments show that the proposed system can support repeated assembly, improve post-assembly accuracy, and accomplish grasp-related tasks with stable execution performance. These results demonstrate the feasibility of integrating modular design, calibration, and task execution in a connected validation framework.

The current study was conducted on a prototype-scale platform, and the experiments mainly verified the feasibility of the proposed mechanical design, calibration framework, and grasping method. Future work will include more extensive repeated experiments and broader cross-platform comparisons to further evaluate the robustness and generality of the proposed framework.

## Figures and Tables

**Table 1:** Comparison of representative existing studies and the present work.

Category	Representative Studies	Main Focus	Limitations with Respect to Modular/Reconfigurable CDPRs	Relation to This Work
Modular mechanical design	[20,35,36,37]	Rapid deployment, reconfigurable structures, or interchangeable end-effectors for task execution.	Most studies mainly emphasize mechanical modularity or application-oriented deployment, while the integration of modular structure with onboard sensing, cable-guiding stability, and anti-slack operation is less discussed.	This work develops an MCDPR prototype integrating drive, sensing, cable-guiding, and anti-slack functions within the same modular framework.
Kinematic modeling	[8,9,10,11,12,13,14,15,16]	Forward/inverse kinematics, static analysis, and numerical or analytical solutions for CDPRs.	Most modeling methods are established for fixed robot configurations, and the influence of repeated assembly or modular reconstruction on the modeling process is rarely considered.	This work establishes a pulley-considered multilayer kinematic model for a modularly assembled MCDPR.
Calibration/self-calibration	[24,25,26,27,28,29,30,31,32,33]	Use of external instruments or onboard sensors to identify geometric parameters and improve robot accuracy.	Existing methods often depend on external measuring devices or are designed for specific robot configurations, which limits their suitability for repeatedly reconfigured modular systems.	This work proposes a vision-based self-calibration method using onboard sensing and scene markers for repeatedly reconfigured modular systems.
Vision-guided grasping/task execution	[34,35,36]	Task-oriented CDPR applications, such as installation or cleaning, with emphasis on application feasibility.	The connection between calibration results and downstream task execution is usually not explicitly established, especially for modular CDPR systems.	This work combines RGB-D visual perception with the calibrated robot model so that calibration results can be directly used for vision-guided grasping.

**Table 2:** Variable definitions.

Symbols	Physical Meaning
Biw	Position vector of the connection point of the *i*th cable to the pulley in the global frame
Aim	Position vector of the connection point of the *i*th cable with the moving platform in the coordinate system of the moving platform
Aiw	Position vector of the connection point of the *i*th cable with the moving platform in the global coordinate system
Pmw	Position vector of the center point (i.e., $O_{p}$) of the moving platform in the world frame
Liw	Length vector of the *i*th cable in the global frame
Lim	Length vector of the *i*th cable in the dynamic frame
$l_{i}$	Length of the *i*th cable
L	The cable length matrix of MCDPRs
Hmw	The homogeneous transformation matrix of the dynamic frame with respect to the global frame
$T_{i}$	The cable tension matrix of MCDPRs
$T_{ci}$	Tension magnitude of the *i*th cable
$T_{c}$	The cable tension matrix for MCDPRs
$D_{c}$	Number of cables in MCDPRs
$D_{m}$	Number of DOFs for MCDPR mobile platforms

**Table 3:** The self-calibration results of the MCDPR.

Variables		True Value (mm/°)	Calibration Value (mm/°)
B1w		[−4.9, −399.6, 24.5]	[−5.003, −399.598, 24.487]
B2w		[40.1, 405.4, 24.5]	[40.089, 405.398, 24.505]
B3w		[637.1, 400.4, 4.5]	[636.987, 400.410, 4.489]
B4w		[632.1, −406.4, 4.5]	[632.109, −406.396, 4.495]
A1m		[−45.0, −65.0, 0]	\
A2m		[−45.0, 65.0, 0]	\
A3m		[45.0, 65.0, 0]	\
A4m		[45.0, −65.0, 0]	\
$\bar{l_{1}}$		403.7	403.686
$\bar{l_{2}}$		426.4	426.409
$\bar{l_{3}}$		231.7	231.693
$\bar{l_{4}}$		428.3	428.294
Pmv	Position	[5.6, 49.8, −31.4]	[5.602, 49.798, −31.395]
	Attitude	[180°, 0, 90°]	[180.011°, 0.002, 90.012°]
Ptw	Position	[320.0, 0, −694.9]	[319.994, 0.005, −694.886]
	Attitude	[−180°, 0, 30°]	[−179.996, 0.003, 29.998]

**Table 4:** The pose of the moving platform before and after calibration.

Name	Expected Pose	Pose Before Calibration	Pose After Calibration
$x_{m}$/mm	355	355.313	354.977
$y_{m}$/mm	−334	−333.896	−334.042
$z_{m}$/mm	−535	−534.831	−534.993
$\gamma_{m}$/°	110.982	111.053	110.978

**Table 5:** Self-calibration results of an MCDPR.

Variables		Theoretical Value (mm)	Calibration Value (mm/°)
B1w		[25.000, −370.000, 24.000]	[25.102, −370.603, 24.100]
B2w		[40.000, 385.000, 24.000]	[40.201, 384.903, 24.200]
B3w		[645.000, 370.000, 24.000]	[644.503, 370.101, 24.500]
B4w		[630.000,−385.000, 24.000]	[629.502, −385.401, 23.900]
$\bar{l_{1}}$		\	368.902
$\bar{l_{2}}$		\	421.309
$\bar{l_{3}}$		\	463.810
$\bar{l_{4}}$		\	412.002
Pmv	Position	\	[22.100, 54.801, −7.502]
	Attitude		[179.909, 0, 89.949]

**Table 6:** Pose errors before and after self-calibration.

Name		Measure Before Calibration (mm/°)	Measure After Calibration (mm/°)
Starting point	Position	[106, 10, 222]	[105.241, 11.226, 223.019]
	Attitude	[180, 0, 90]	[179.109, 0.998, 90.328]
End point	Position	[−68, 13, 232]	[−67.219, 12.834, 232.982]
	Attitude	[−180°, 0, 90°]	[−179.286°, 1.028°, 90.463°]

**Table 7:** The parameters of the circular trajectory.

Starting Point Pstartw (mm)	Motion Center Point Pcenterw (mm)	The Radius (mm)
[255.0, −105.0, 24.0]	[335.0, 0, 24.0]	65

**Table 8:** Cable length error.

Starting Point (m)	End Point (m)	Average Cable Length Error (m)	Maximum Cable Length Error (m)
(0.5, 0.2, 0.024)	(0.2, −0.2, 0.024)	(0.0014, 0.0016, 0.0004)	(0.0029, 0.0031,0.0010)

## Data Availability

Data is contained within the article. Further inquiries can be directed to the corresponding authors.
